# Implementing individual placement and support in autistic and other intellectually and developmentally disabled adults: lessons learned in California

**DOI:** 10.3389/ijph.2026.1610040

**Published:** 2026-08-12

**Authors:** Marjorie Solomon, Jo Ann Yon-Hernández, Megan M. Smith, Maggie Zheng, Christopher Llorente, Amanda Downing, Steve Ruder, Aubyn C. Stahmer, Susan R. McGurk

**Affiliations:** 1 Department of Psychiatry and Behavioral Sciences, MIND Institute, University of California, Davis, Sacramento, CA, United States; 2 Alameda County Behavioral Health Department, Oakland, CA, United States; 3 Mental Health Systems, San Diego, CA, United States; 4 Center for Psychiatric Rehabilitation, Boston University, Boston, MA, United States

**Keywords:** individual placement and support, intellectual disability, adults, autism, developmental disabilities, employment, service system

## Introduction

Autistic young adults enter the workforce at lower rates than their non-autistic peers, and this has a profound impact on their wellbeing and life satisfaction [1]. To address this problem, in the U.S., close to twenty states have eliminated the subminimum wages which encourage the continued use of segregated sheltered workshops for individuals with intellectual and developmental disabilities, including autistic people (collectively referred to as individuals with IDD). Despite this change, the most recent California statistics suggest that only 13% of persons with IDD receiving state services work in paid jobs. Furthermore, many of these jobs are not in truly competitive integrated employment (CIE) (i.e., jobs with comparable pay, benefits, integration, and advancement opportunities to workers without disabilities) [2]).

To address the problem of unemployment and under-employment of adults with IDD and capitalize on opportunities for change created by the elimination of sheltered workshops, we now are conducting a second trial of an adaptation of Individual Placement and Support (IPS) – the most highly evidence-based supported employment model [3] – in California [4]. In this Commentary, we discuss our experience as a model of how policy, systems and health service factors influence the implementation of an evidence-based practice in our state and potentially others. We begin by discussing the systemic differences in service delivery between the IDD and mental/behavioral health service systems. We then review the compatibility of the California supported employment system for individuals with IDD and the eight core principles of IPS. Finally, we describe challenges in implementing IPS fidelity measures for IDD.

## Service delivery system

California’s state-supported IDD service system is directed by the Department of Developmental Services and implemented by 21 Regional Centers [5]. For eligible consumers/clients, Regional Centers provide case management by a service coordinator, who typically has more than 100 clients. Service coordinators direct purchases from vendors providing speech and language, social skills, respite care, mental health provider, independent living, and multiple vocational services. When Regional Center clients become young adults and express interest in working, they must first be referred to the California Department of Rehabilitation. This Department provides career assessments and uses a milestone-based job acquisition approach that pays vocational service providers in fixed installments based on progress toward and retention of CIE. Clients who have not attained employment and need additional job development or support can be re-referred to their Regional Center for assistance as long as they want and need. IDD service providers attempting high-fidelity IPS, which involves both job development and ongoing job support, often face the complex task of creatively “braiding” funding using reimbursements from both State Agencies.

California is not unique in having multiple service systems related to IDD and vocational services, and coordination between these systems can vary greatly by region in terms of complexity and level of available resources. It also bears mention that in California, unlike a few states with better service integration, the mental health system is not routinely involved with clients entering the system through the IDD pathway. For most IDD clients, mental health professionals are vendors, referrals are provided as needed, and there is limited integration between vocational and behavioral/mental health teams.

## Compatibility between employment services for adults with IDD and IPS principles

Employment practices implemented by the California services system are compatible with the eight core principles of IPS to varying degrees (Table 1). For example, California’s overall employment approach stresses respect for client preferences and CIE, because it is based on the federal Workforce Innovation and Opportunity Act legislation, which has been implemented and enforced to different degrees in different states. Consistent with IPS principles, under the Lanterman Act, Regional Centers also are required to provide individualized, long-term follow-along support that continue for as long as the client wants and needs.

**TABLE 1:** Individual placement and support (IPS) principles and their compatibility with the California developmental disabilities system (California, United States. 2026).

IPS principle	Compatibility with California practices	Implementation feasibility	Contextual appropriateness	Comments
Competitive employment	High	High	High	California is an employment first state
Client preferences	High	High	High	California is an employment first state
Time-unlimited support	High	High	High	Funding supported by RCs
Rapid job search	Low/Medium	Low/Medium	High	Not a consistent agency practice
Systematic job development	Low/Medium	Low/Medium	High	Not a consistent agency practice
Benefits counseling	Low/Medium	Low/Medium	High	Not a consistent agency practice
Integrated with mental health	Low	Low	Low	Not part of the DD system
Zero exclusion	Low	Low/Medium	Low	People with high support needs may not be able to work safely in all settings; however, exclusion should be highly limited

RCs, Regional Centers; DD, Developmental Disabilities.

Although IPS principles of rapid job search, systematic job development involving the creation of strong employer relationships through repeated community contact, and routine provision of benefits counseling to clients have been validated for consumers served in the behavioral/mental health system [6], these principles are not always implemented in IDD employment services. Given requirements to utilize Rehabilitation Department services first, clients often must participate in lengthy assessments and training before jobs are developed. Additionally, employment specialists frequently lack formal training in best practices [7]; find it relatively easier to evaluate and place clients in training programs compared with engaging in community-based job development; and have ableist and paternalistic views that underestimate consumer job readiness. Furthermore, reimbursement policies may tacitly encourage overuse of unpaid work experiences, internships, and classes. These last issues may be present in other states and contexts.

Several other IPS principles are more difficult to implement in our state given how agencies typically deliver services and IDD consumer characteristics. The greatest challenge is service integration. Because most clients are not regularly served by mental health providers and service coordinators have such large caseloads, interdisciplinary team meetings are not used. A second challenge involves the IPS zero exclusion principle, given that some IDD consumers have high support needs, severe intellectual disabilities, minimal verbal communication, and/or safety needs that preclude traditional jobs. Finally, some agencies implicitly sort clients by who they believe can and cannot work and/or are unduly pressured by parents wanting their children placed in non-beneficial internships. Both practices violate zero exclusion. Again, they are likely not only a California problem.

## Challenges in fidelity reviews

IPS relies on an empirically validated fidelity review scale and process. As described above, however, IDD service system agencies typically have difficulty scoring at the highest levels (i.e., higher than “Good”) because integrated teams including mental health providers are not funded or used. The existence of such IDD-mental health silos is true in most states. To address such service integration problems, some agencies that include a broader range of independent living or school-based services, engage in regular meetings and function as an integrated multidisciplinary team, as has been contemplated by the IPS-Y Fidelity Scale for Young Adults [8].

In conclusion, we believe the core structure of IPS is well-suited to helping consumers with IDD, and that this more manualized and evidence-based supported employment model holds the potential to greatly improve service quality, employment outcomes, and consumer quality of life in many states. However, in California and other states, there are significant barriers to implementation due to philosophies about how to best improve employment outcomes, and how supported employment agencies operate and are reimbursed. In California, we would point out the behavioral/mental health system has set a transformative precedent: under Proposition 1 and the Behavioral Health Services Act of 2024, high-fidelity IPS is now a mandatory mental health standard of care across all counties [6]. The IDD service system may stand at a critical crossroads, whereby State Agencies that provide IDD services could leverage this momentum of the mental health system to modernize their practices. We have seen that IPS principles of leadership engagement, reinforced by the fidelity review feedback and planning processes, provides a mechanism for promoting organizational change in the agencies we are working with. This provides hope that with the help of their advocacy, results of our empirical work, and State Agency support, the time may be right for meaningful improvements in IDD employment services in California.
